# The Challenge of Weight Stigma for Women in the Preconception Period: Workshop Recommendations for Action from the 5th European Conference on Preconception Health and Care

**DOI:** 10.3390/ijerph20227034

**Published:** 2023-11-08

**Authors:** Briony Hill, Alexandra Azzari Wynn-Jones, Kimberley J. Botting, Emma H. Cassinelli, Michael P. Daly, Caitlin Victoria Gardiner, Stephanie J. Hanley, Nicola Heslehurst, Regine Steegers-Theunissen, Sarah Verbiest, Helen Skouteris

**Affiliations:** 1 Health and Social Care Unit, School of Public Health and Preventive Medicine, Monash University, 553 St Kilda Rd, Melbourne 3004, Australia; 2 Independent Researcher, Henley-on-Thames RG9 1HG, UK; hello@thehealthandwellbeingnutri.com; 3 Department of Maternal and Fetal Medicine, Elizabeth Garrett Anderson Institute for Women’s Health, University College London, London WC1E 6HX, UK; k.botting@ucl.ac.uk; 4 Centre for Public Health, School of Medicine, Dentistry and Biomedical Sciences, Queen’s University Belfast, Belfast BT12 6BA, UK; ecassinelli01@qub.ac.uk; 5 Centre for Public Health, Bristol Medical School, University of Bristol, Canynge Hall, Whatley Road, Bristol BS8 2PN, UK; michael.daly@bristol.ac.uk; 6 Department of Global Health and Social Medicine, Bush House, Strand Campus, King’s College London, 40 Aldwych, London WC2B 4BG, UK; caitlin.gardiner@kcl.ac.uk; 7 Developmental Pathways for Health Research Unit, University of the Witwatersrand Faculty of Health Sciences, Johannesburg 2000, Gauteng, South Africa; 8 Institute of Applied Health Research, University of Birmingham, Edgbaston, Birmingham B15 2TT, UK; s.hanley@bham.ac.uk; 9 Population Health Sciences Institute, Faculty of Medical Sciences, Newcastle University, Newcastle upon Tyne NE2 4AX, UK; nicola.heslehurst@ncl.ac.uk; 10 Department of Obstetrics and Gynaecology, and Department of and Pediatrics, Erasmus MC, University Medical Center, 3015 GD Rotterdam, The Netherlands; r.steegers@erasmusmc.nl; 11 School of Social Work, University of North Carolina at Chapel Hill, Pittsboro Road, Chapel Hill, NC 27599-3550, USA; sarah_verbiest@med.unc.edu; 12 Warwick Business School, University of Warwick, Scarman Rd, Coventry CV4 7AL, UK

**Keywords:** weight stigma, preconception, healthcare, obesity, recommendations

## Abstract

Weight stigma is a well-recognised public health issue affecting many members of society including women during the preconception period. The impacts of preconception weight stigma on women are significant and may result in decreased access to and uptake of healthcare, and mental health concerns. The consequences of this weight stigma may translate to negative maternal outcomes and even intergenerational effects on the child. Eliminating weight stigma is therefore imperative. The aim of this paper is to report recommendations to reduce weight stigma for preconception women produced at a workshop with clinical and academic experts on preconception health and weight stigma at the 5th European Conference on Preconception Health and Care. The recommendations are related to two key areas: general societal recommendations prompting all people to acknowledge and adjust our attitudes towards larger-bodied people; and healthcare-specific recommendations imploring clinicians to upskill themselves to reduce weight stigma in practice. We therefore call for urgent approaches to address societal weight-stigmatising attitudes and norms related to both the general population and preconception women, while providing professional development opportunities for healthcare professionals relating to weight stigma. Eliminating weight stigma for preconception women may have positive impacts on the outcomes for mothers and children during pregnancy and beyond.

## 1. Introduction

Weight stigma is a pervasive stereotypical and discriminatory perception that being larger-bodied is inherently pathological, and that individuals are solely responsible—and thus subject to judgement and blame—for their own weight [1,2]. Weight stigma is associated with the view that a particular body size is more valuable or worthy than another and remains a human rights issue [3,4]. In those who experience it, weight stigma can contribute to poor mental health and psychosocial wellbeing, reduced uptake of healthcare services, and suboptimal diet and physical activity behaviours, which reinforce or increase both higher body weight and weight-based stigma [5,6,7]. 

Weight stigma and discrimination can be issues for people of all ages [6]. However, women may experience unique, additional sources of weight stigma and discrimination. Indeed, women of reproductive age (while some pregnant people may not identify as women, the current literature base to date has largely focused on the views of women) report experiencing up to three weight-stigmatising events per day [8]. Weight stigma experienced in the reproductive years also contributes to a raft of negative consequences specific to this life stage, including poor uptake of reproductive healthcare, adverse pregnancy outcomes (including mental health concerns), suboptimal birth outcomes, and poorer long-term maternal and child health [8,9]. This weight stigma can arise due to prevailing perceptions within society that a woman’s body is simply a vessel to carry a baby and that any excess weight signifies significant risks to the baby [10,11]. This blame extends from the wider literature suggesting that preconception and pregnancy environments can influence the wellbeing and development of future generations, for example, through epigenetic changes [12], and biomedical discourse that legitimises controlling women’s bodies [13]. This intersects with (predominantly Western) societal expectations for women to attain a thin and thus more ‘attractive’ body [14,15]. It is also possible that the extensive research into maternal obesity and the comparatively limited evidence for the contribution of paternal obesity to offspring outcomes [16] have led to an unbalanced perspective in the media that reinforces weight stigma and related messages for women [17].

Weight stigma is most prevalent in women, particularly those of reproductive age [18]. This age range aligns with common interpretations of the preconception period [11,19]. Preconception can be defined in multiple ways, including a lifecourse approach that recognises the importance of preconception health for all, a public health view that incorporates all people of reproductive age and recognises the prevalence of unplanned pregnancies, as well as a more targeted view of people who are consciously planning a pregnancy or trying to conceive [19]. Few studies have investigated weight stigma and weight-based discrimination explicitly in the preconception period [8]. The limited empirical evidence available suggests that women with larger bodies are shamed for wanting to conceive and may be denied fertility treatment until they have lost weight [20]. Aligned with this, health services and providers in countries such as the United Kingdom [21], Australia [22], and the United States [23] have recommendations that limit eligibility for fertility treatment to women with body mass indices below 30 kg/m^2^ [22,23], or even 25 kg/m^2^ [21]. Weight stigma can also prevent healthcare professionals from identifying underlying health conditions that could be contributing to weight gain, as well as other health issues that are important to address such as diabetes, cardiovascular and endocrine problems, and medication side effects [24]. This becomes particularly important for women with chronic conditions who subsequently experience delays in diagnosis and treatment because they are foremost told to lose weight [25,26]. Women are also susceptible to internalised weight bias which may manifest as self-blame if they are struggling to conceive or if they have experienced pregnancy loss [27,28].

Weight stigmatisation is an ubiquitous experience for larger-bodied women of reproductive age, extending across healthcare, society and community, peers, family, and the media [8]. Addressing weight stigma is, therefore, a relevant issue for individuals, communities, and governments. In addition to affecting women’s health before and at conception, weight stigma in the preconception period may also affect the health of future generations [8]. We (the authors) attended the 5th European Conference on Preconception Health and Care in September 2022. We observed that weight stigma is an area gaining increasing attention, yet with little evidence available to inform action in the preconception health arena. As a formal part of the congress, authors BH and HS conducted a workshop on the topic of weight stigma by engaging attendees in a discussion on how to reduce or eliminate weight stigma and discrimination against women during the preconception period. The aim of this paper is to report the findings of the workshop via meaningful recommendations to reduce preconception weight stigma with the aim of contributing towards an ultimate goal of eradicating weight stigma for preconception women.

## 2. Materials and Methods

Authors BH and HS led a 45-min workshop at the 5th European Conference on Preconception Health and Care. The stated focus of the workshop was reframing the preconception “lifestyle health” narrative for women from one that focuses predominantly on individual responsibility for diet, physical activity, and weight management practices to one that transcends individual blame and weight stigma by addressing the broader socioecological determinants of health and body size. Eleven other conference delegates attended the workshop, including eight in-person and three online via Zoom as per the conference’s hybrid format. Participants all had an interest in preconception weight stigma and included clinicians and academic researchers from Australia, Ireland, Japan, The Netherlands, South Africa, the United Kingdom, and the United States. All were PhD-qualified, with the exception of one participant who had a Master’s degree. Research expertise included pre-clinical studies, bioethics, intervention development, maternity services, health and developmental psychology, nutrition, public health, preconception health, maternal obesity, and weight stigma. Two participants were qualified Medical Doctors, and one was a public health expert.

Workshop facilitators presented for 15 min on the concept of weight stigma and its relevance for preconception women, including: women’s experiences of weight stigma; the factors that drive and enable weight stigma; where it occurs; and its deleterious impacts. All workshop content was supported by evidence reviews [8]; key content is presented in Table 1. The workshop focused on women, acknowledging that gender-diverse people experience a range of intersecting stigmas that impact their weight stigma experience [29,30]. 

After the initial background presentation, participants completed a 30-min brainstorming activity in two groups (one online and one in-person). For this, they were asked to generate actionable ideas to reduce weight stigma and discrimination for preconception women from both an individual perspective (i.e., what can individuals do?) and a societal perspective (i.e., what can society do?). The prompt question was, “how can we move past individual blame and discrimination and reduce weight stigma for preconception women?”. Workshop participants were not given direction on how to interpret the definition of preconception. Participant discussions were captured by a nominated scribe and shared back to the full group at the end of the session. 

After the workshop, the lead author/facilitator (BH) summarised the key discussion points into recommendations and collated them into naturally occurring themes. These recommendations were discussed with the senior author/facilitator (HS) until an agreement was reached. Subsequently, the recommendations were circulated via email and reviewed by all workshop participants. A lively asynchronous email discussion of the content ensued before attempting to achieve group consensus on the recommendations arising from the workshop. Our key recommendations relate to areas where group consensus was reached, and a critical discussion of additional factors is presented where group consensus was not reached.

## 3. Results

The recommendations that were developed from the workshop discussions fit into two key areas (i.e., topic themes): (1) general societal recommendations—“acknowledge and adjust our attitudes”; and (2) healthcare-specific recommendations—“upskill, consider, and include” (Table 2). The healthcare-specific recommendations primarily focus on what healthcare professionals can do (as individual clinicians) to reduce weight stigma. While the workshop and recommendations were aimed specifically at women in the preconception period, it was recognised that many recommendations are relevant to all genders across the life course.

The theme “acknowledge and adjust our attitudes” incorporated recommendations that centred on the need for many small and large adjustments to our attitudes as a society to address preconception weight stigma. These general societal recommendations included acknowledging the complex and multifactorial nature of weight/body size/obesity with the intent of moving away from moral judgement and the pathologisation of larger bodies. These recommendations speak to well-established evidence highlighting the socioecological determinants of body size, including both environmental and genetic factors [31,32]. Listening to the voice of people with lived experience of living in larger bodies was a unanimous recommendation that is supported by evidence [33] and funding bodies across the globe [34,35,36]. Discussions also highlighted the importance of consciously challenging harmful media perspectives that are perpetuated against larger-bodied people, such as the common use of headless images of larger bodies and sensationalised and stigmatising headlines [37,38]. This was closely aligned with the recommendation to continue to improve the discourse around obesity by using non-stigmatising images and language, particularly in healthcare settings and public places such as websites and social media. The group also acknowledged a growing awareness of intersectionality and body size stigma. For example, we discussed increased stigma for women of colour with larger bodies in certain geographies as well as the inequalities associated with socioeconomic position, education, and gender that may exacerbate weight stigma. 

The theme “upskill, consider, and include” centred on healthcare-specific recommendations. Discussions trended to preconception and fertility care and were primarily focused on the possibility of clinicians making individual changes to their practice. For example, further consideration of patient needs in relation to weight stigma could improve the sensitivity of conversations with patients who are living in larger bodies. Specific recommendations included the need to provide clinicians with professional development opportunities and training to understand the power of their words and to be mindful of patient needs and feelings. This recommendation was closely aligned with a call for consultations to be re-focused away from weight, where appropriate, and focus the conversation on patients’ general health and wellbeing [30]. This was to avoid perpetuating a blame narrative for women, especially when wanting to start a family. Similarly, participants highlighted that the communication of relative risks (compared to absolute risk) may exaggerate the potential for adverse outcomes related to weight or body size. The Five A’s tool (Ask, Advise, Assess, Assist, and Arrange) [39] was suggested as a useful way for clinicians to prioritise patient needs and remain patient-centred, thereby limiting the potential for their own weight biases to affect patient care. Lastly, as with the general societal recommendations, workshop attendees agreed that the views of people with lived experience (e.g., patients/consumers, public/community) should be incorporated by involving them in the co-design of future research, health services, and policies. 

One point of discussion that was not a recommendation but deserves merit was the value of the non-academic literature in recognising body diversity. Specifically, discussions arose around resources that participants had critically evaluated and believed to be valuable for changing the blame narrative. For instance, attendees noted how “body positive” websites and social media (i.e., resources aimed at celebrating the multifaceted nature of positive body image such as body appreciation and acceptance [40,41]) could influence community and individual attitudes towards people with larger bodies for the better. Indeed, it has been argued that social media has the potential power to promote public awareness and policy change regarding weight stigma [42]. However, we also acknowledge that further research is required to evaluate both the positive and negative impact of body-positive social media on weight stigma, particularly internalised weight biases, and its potential for reinforcing negative stereotypes [43]. 

There were two points of contention that attracted critical discussions. One of these related to whether we should recognise obesity as a disease, as there is not yet agreement from the academic or medical community on this issue [44]. On one hand, there was some support for the framing of obesity as a disease, for which a precedent has been set. For example, obesity was first included in the International Classification of Diseases in 1948, and subsequently defined as a “complex multifactorial disease defined by excessive adiposity and is linked to an increased risk for many non-communicable diseases” by the World Health Organization [45](p. 2), Parliaments in some countries (e.g., Portugal, 2004; Italy, 2019 [46,47]), and the European Parliament in 2021 [48]. The ‘obesity as a disease’ perspective highlights the need to recognise human rights relating to people having larger bodies and to create a requirement for evidence-based prevention and treatment strategies. In the context of stigma, there are arguments that these actions focus the narrative away from the individual blame, responsibility, and negative stereotypes that contribute towards stigma, and promote demand for weight being recognised as a protected characteristic to protect from discrimination [49]. This approach has also been advocated by some patient organisations as a strategy to address stigma directly [50,51].

On the other hand, while all did agree that there is a need to move away from individual moral judgement and blame around obesity, there was a counter argument that labelling obesity as a disease should be approached with caution. This argument relates to a view that framing obesity as a disease further pathologises having a larger body. This can be problematic; having a larger body is readily visually perceived by others, and thus the label of ‘diseased’ or ‘unhealthy’ may instead be attributed to these persons, even when their larger body size is not pathological. Thus, while an individual’s blameworthiness is reduced, essentialising obesity as an enduring component of a person’s identity can heighten stigma and prejudice [52,53]. Obesity’s pathologisation may also increase weight discrimination in the healthcare setting, especially if it becomes a (more legitimised) primary focus of the clinical encounter to the exclusion of a patient’s other health issues [54,55]. Additionally, there are still significant challenges in defining and measuring obesity [49,56,57]. Although the definition of obesity as a disease is not based on body mass index, body mass index is regularly used in policy and practice to define obesity in relation to the risks associated with body size [58]. This approach risks homogenising all people with larger bodies. There is an argument that this may also promote a focus on individualised pharmacological and surgical solutions, thereby circumventing preceding social, structural, and environmental factors that are significant contributors to the development of larger body size [59,60,61]. However, it is important to note that evidence-based treatments should be made available to those who want/need them, in addition to addressing upstream determinants as part of a strategy to optimise population health. 

A second area of contention in discussions was the common use of the term “lifestyle”. On one hand, the term enables one to move discussions around obesity towards general health. It is often used as an “encompassing” term to include weight-related behaviours (e.g., diet and physical activity), and can allow attention to be directed at individual agency to control our own health [62]. While the participants did not dispute that behaviours are important to consider in the context of weight, there was a view by some that the term “lifestyle” focuses solely on the individual and ignores other important determinants. Framing weight in the context of lifestyle, in turn, may promote weight stigma by reinforcing a narrative that individuals with higher weight have made the wrong behavioural choices, lack self-control, and are personally responsible and blamed for their weight, rather than shifting the narrative to the upstream social, environmental, and commercial determinants of health [62]. After discussion, we elected to avoid using the term “lifestyle” in our recommendations to ensure that we are not inadvertently contributing to weight stigma. However, we recognise the need to empower women to have choice and control in the way they live. This is especially important for women planning pregnancy where many other choices are taken away (e.g., what to eat) and the focus shifts to optimising the body for a healthy pregnancy [10].

## 4. Discussion

The aim of this paper was to present meaningful recommendations to reduce weight stigma in the preconception period as identified at our workshop. This represents the first attempt, to our knowledge, to voice recommendations to improve the treatment of larger-bodied people in the preconception period. Recommendations fit into two key topic themes: (1) general societal recommendations—“acknowledge and adjust our attitudes”; and (2) healthcare-specific recommendations focusing on clinician behaviour—“upskill, consider, and include”. Many of the discussion points stemmed from the workshop participants’ knowledge of the weight stigma literature, but others evolved from participants’ broader experiences, practice, and prior research. There were also issues of contention, such as defining obesity as a disease and the common use of the term “lifestyle”.

Acknowledging and addressing societal attitudes towards women living in larger bodies is receiving increasing attention [8,63]. Whilst the recommendations developed from the workshop were focused on changes to collective societal thinking about people in larger bodies (e.g., acknowledge the complex, multifactorial nature of body weight; subvert harmful media messages), we recognise that approaches to address this are complex and multifaceted. Indeed, discussions across both general and healthcare-specific recommendations repeatedly acknowledged that structural societal changes are needed to reduce systemic bias, weight stigma, and discrimination alongside targeting individuals who might be perpetuating stigma. An example from the UK with global relevance was the identification of the factors related to environmental, fiscal, and political drivers of obesity that are outside of individuals’ “lifestyle choices”. These include unacceptably high levels of food insecurity that disproportionately affect families with young children [64]; record levels of food inflation and increasing energy costs globally [65]; and cuts to public health spending being the highest in the most deprived locations [66].

Societal attitudes towards women living in larger bodies are perpetuated by stigmatising public health campaigns promoted through the media that flaunt the narrative of individual responsibility for developing obesity and losing weight, ignoring (or not appropriately addressing) the wider causes and contributors to obesity [67,68]. These public health messages heavily influence public sentiment towards larger-bodied individuals [37,69]. These issues affect the broader population and reflect a lifecourse preconception perspective, a perspective for defining preconception that is inclusive of the periods of adolescence and young adulthood and which spans women’s lives in general [19,70]. However, the issues are particularly salient in the immediate preconception period due to the potential for intergenerational impacts on offspring [8]. For instance, while evidence has not yet been generated for the intergenerational transmission of weight stigma [71], compared to women who do not experience weight stigma, the deleterious impacts of weight stigma such as depression [18], body image concerns [72], and decreased engagement with preconception healthcare are known to negatively impact pregnancy and offspring outcomes [73,74]. Furthermore, because people with larger bodies have restricted access to fertility treatment [20] and may experience fiscal and policy-related barriers to optimising diet and physical activity behaviours [66], their agency for their own health is impacted by their experiences of stigma [8]. This presents the paradoxical situation of further reducing their capability to lose weight and reinforcing weight stigma experiences.

There is much that healthcare professionals working with preconception women can do to address weight stigma. For example, sensitivity around weight-related discussions was noted as particularly important, especially for women planning pregnancy. Healthcare professionals, including midwives and general practitioners, consistently report a lack of confidence and skills in having conversations around weight [75,76]. However, training can improve these skills [77]. The Five As tool, a counselling technique for smoking cessation, has been successfully modified for obesity counselling and addresses stigma in healthcare by transferring agency to the patient about whether their weight is a topic they are willing to discuss at that particular healthcare visit [78]. As part of this, we can also ensure that conversations around weight are moved from a focus on weight loss and body size to one of health processes (i.e., positive health behaviours and actions) [79]. These strategies can assist with maintaining patient-centred care that avoids a “blame narrative” and can be strengthened by incorporating patients’ experiences and views in the co-design of health services and medical education [80,81]. There are prospects here for public health advocates to produce professional development opportunities for clinicians.

Our proposed recommendations for eliminating preconception weight stigma are fundamentally aligned with recommendations to reduce weight stigma more generally, such as the Joint International Consensus Statement for Ending Stigma of Obesity [3]. The Ending Stigma of Obesity statement highlights that weight-based stigma should not be tolerated, recognises the flawed conventional narrative around obesity, and makes specific recommendations for healthcare professionals, including the introduction of appropriate infrastructure to ensure stigma-free practice. While broad stigma reduction strategies are useful, our recommendations for reducing preconception weight stigma should be considered salient because they acknowledge the unique needs of women who are attempting to conceive. This includes women seeking assisted reproductive services and recognises that many women do not disclose pregnancy plans due to the stigma around miscarriage. We also advocate to avoid perpetuating the “mother blame” narrative from before conception has even occurred and mitigating the potential impact of weight stigmatising experiences on uptake and engagement with preconception healthcare. 

We also retained some areas of discussion where a consensus could not be reached. These were the topics of framing obesity as a disease and the use of the word “lifestyle”. These concerns clearly have ramifications which extend beyond the preconception period and are receiving significant debate in both the broader media and academic literature [62,82,83]. One area we did agree on at the workshop was that engagement with multiple stakeholders is required to explore these complex topics.

### Limitations and Strengths

Our workshop was limited to 45 min, meaning that the breadth and depth of discussions may have been impacted. However, we aimed to overcome this by continued, asynchronous post-workshop discussions before arriving at the final list of recommendations presented in this paper. The workshop was attended by delegates of a preconception health conference, and hence, perspectives from experts in other relevant topic areas such as social science, as well as those of people with lived experience, may not have been captured. Nevertheless, the participants represented an impressive range of expertise including bioethics, public health, maternity services, psychology, nutrition, preconception health, maternal obesity, and weight stigma. Our workshop also included perspectives from medical doctors. We also achieved participant representation from seven countries. While the focus of this paper is on women in larger bodies, we also recognise that weight stigma may be perpetuated against people in low-weight bodies, individuals with eating disorders [84], and people in other sex and gender categories including men and non-cisgender persons; the unique experiences of these groups relating to weight stigma also merit attention [85].

## 5. Conclusions

Weight stigma in the preconception period is a largely overlooked area in need of urgent attention to reduce adverse stigma-related outcomes for women and the next generation, including compromised engagement in preconception care. We call for specific attention to the preconception period to avoid perpetuating the intergenerational transmission of the individual responsibility and blame narrative for women living in larger bodies. We recommend that public health advocates aim to address weight-stigmatising attitudes and societal norms, while healthcare professionals working with women in the preconception period should seek upskilling opportunities around weight stigma and focus on being sensitive to the unique needs of women in larger bodies. These recommendations focus on the need to transcend individual blame and responsibility for the complex issue of weight, and seek multi-faceted solutions. Eliminating weight stigma for preconception women may have positive onflow impacts on the outcomes for mothers and children during pregnancy and beyond.

## Figures and Tables

**Table 1:** Workshop presentation content *.

Workshop Topic	Detail
Where preconception weight stigma occurs	People and settings in the wider community Media Family, friends, and other mothers Workplaces Healthcare
Women’s experience of weight stigma	Feeling hurt, judged, shamed, embarrassed, and humiliated Made to feel individually responsible for problems that arise during pregnancy Assumptions made about a person—diet/physical activity behaviours, laziness, or lack of self-control
Stigma marking and intersecting stigmas	Body mass index, prior gestational weight gain, postpartum weight retention Gender Race/ethnicity
Factors that drive and enable weight stigma	Stereotyping Negative interactions with healthcare providers Biased assumptions about the causes and consequences of obesity Poorly considered policies Refusal of care Inappropriate, devaluing, and negative comments
Known and potential health and social impacts, including transgenerational consequences	Decreased access to and uptake of reproductive healthcare Decreased psychosocial wellbeing Poorer motivation and self-efficacy for health behaviours Poorer pregnancy and birth outcomes Adverse impact on breastfeeding Poorer longer-term mother and child outcomes

* Content derived from Hill and Incollingo Rodriguez, 2020 [8].

**Table 2:** Recommendations to work towards eradicating weight stigma against women in the preconception period.

General societal recommendations—“Acknowledge and adjust our attitudes”
Acknowledge the complex and multifactorial nature of body weight Move away from the moral judgement and pathologisation of larger bodies Listen to the voice of people with experience of living in larger bodies Subvert, not reinforce, harmful media perspectives of people in larger bodies Use non-stigmatising images and language—especially on websites and social media Acknowledge different and varying cultural beliefs around body size
Healthcare-specific recommendations—“Upskill, consider and include”
Upskill clinicians to understand the power of their words and be mindful of patient needs and feelings Reframe the healthcare focus on weight to avoid a “blame narrative” Acknowledge that communicating relative risks may exaggerate the potential for adverse outcomes compared to absolute risk Remember the Five As tool (Ask, Advise, Assess, Assist, and Arrange) to prioritise patient needs Co-develop services with people who have lived experience of being in a larger body

## Data Availability

No new data were created or analysed in this study. Data sharing is not applicable to this article.
